# Gastrodin mitigates aortic valve calcification by inhibiting glycolysis and histone lactylation through interfering with valve interstitial cells

**DOI:** 10.3389/fphar.2025.1547716

**Published:** 2025-06-10

**Authors:** Yu Zhang, Shunshun Wang, Jiaqin Wu, Qianqian Du, Huiming Yu, Li Yang, Xianqiong Liu, Kang Xu, Chunli Wang, Fan Feng

**Affiliations:** ^1^ National Innovation and Attracting Talents “111” Base, Key Laboratory of Biorheological Science and Technology, Ministry of Education, College of Bioengineering, Chongqing University, Chongqing, China; ^2^ Hubei Shizhen Laboratory, Wuhan, China; ^3^ School of Pharmacy, Hubei University of Chinese Medicine, Wuhan, China; ^4^ School of Laboratory Medicine, Hubei University of Chinese Medicine, Wuhan, China

**Keywords:** calcific aortic valve disease, natural product, glycolysis, histone lactylation, gastrodin

## Abstract

Calcific aortic valve disease (CAVD) is the most common disease of the heart valves and is characterised by thickening, fibrosis and calcification of the aortic valve leaflets. Gastrodin, the active component of the traditional Chinese medicine *Gastrodia elata Blume*, has antioxidant, anti-inflammatory, anti-apoptotic and antiviral activities and is widely used in the treatment of neurological and cardiovascular diseases. Here, we report that gastrodin attenuates calcification in CAVD, but the underlying mechanism is unclear. In the present study, we investigated the molecular targets and signaling mechanisms by which gastrodin inhibits CAVD calcification. *In vitro* experiments such as Alizarin Red staining and In-cell western were used to evaluated the anti-calcification effect of gastrodin in the treatment of aortic valves. Transcriptome sequencing and gas chromatography-mass spectrometry analyses showed that gastrodin inhibited the glycolysis level of valvular interstitial cells (VICs). Mechanistically, gastrodin reduces the glycolysis level and lactate production of VICs by inhibiting the enzymatic activity and protein expression of PKM2. Notably, gastrodin treatment inhibited the correlation between histone lactylation H3K9la, a novel lysine-modified modality using lactate as a substrate, and the CAVD marker BMP2. The beneficial effect of gastrodin in reducing aortic valve calcification was demonstrated *in vivo* in high-fat fed ApoE^−/−^ mice. In conclusion, our study shows that gastrodin exerts its anti-calcific effect by interfering with glycolysis and lactylation of VICs, demonstrating the potential of gastrodin as therapeutic agent for CAVD.

## Graphical Abstract

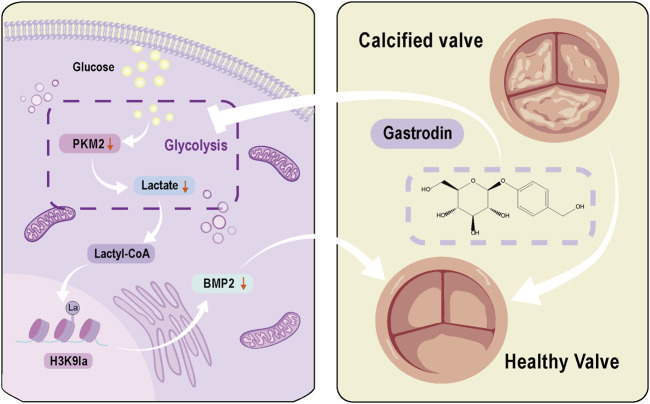

## 1 Introduction

Calcific aortic valve disease (CAVD) represents a significant and growing public health concern (Huang et al., 2023). Characterized by the abnormal accumulation of calcium deposits within the aortic valve leaflets, CAVD leads to progressive stiffening and narrowing of the valve opening, ultimately hindering blood flow from the heart’s left ventricle to the aorta (Blaser et al., 2021). This condition, also known as calcific aortic stenosis (AS), is the most prevalent form of native valve disease, affecting a substantial portion of the elderly population (Wang et al., 2021). The pathogenesis of CAVD is complex and not fully elucidated. However, current understanding suggests a multifactorial process involving chronic inflammation, endothelial dysfunction, lipid modification, and dysregulation of calcium homeostasis within the valve tissue (Coté et al., 2013; Kraler et al., 2022; Ma et al., 2020; Zhou et al., 2020). These factors contribute to a cascade of cellular events, including valvular interstitial cells (VICs) activation and osteogenic transformation, leading to the characteristic calcium build-up and subsequent valve dysfunction (Xu et al., 2020).

In the healthy aortic valve, VICs function as resident fibroblast-like cells, maintaining tissue structure and homeostasis (Cirka et al., 2015). However, various environmental stimuli, including hemodynamic stress and inflammatory signals, can trigger VICs activation (Dayawansa et al., 2022). This activation leads to a phenotypic shift, transforming these cells from quiescent fibroblasts into myofibroblasts and even osteoblast-like cells (Adhikari et al., 2023). These transformed VICs promote excessive production of extracellular matrix components and initiate the abnormal deposition of calcium, ultimately contributing to the characteristic valve dysfunction observed in CAVD (Yuan et al., 2024). This newfound understanding of VICs involvement in CAVD pathogenesis has opened exciting avenues for therapeutic development. By targeting VICs function, researchers aim to prevent their activation, modulate their differentiation pathways, and potentially reverse established calcification (Wang et al., 2025).

Gastrodin is a naturally occurring chemical compound found within the tubers of the *Gastrodia elata Blume*, an herb with a long history of use in Traditional Chinese Medicine (TCM) (Xie et al., 2023). It is classified as a phenolic glycoside, a specific type of molecule with potential health benefits. As the primary bioactive constituent of *Gastrodia elata Blume*, gastrodin has attracted significant scientific interest in recent years due to its diverse pharmacological properties (Xiao et al., 2023). Studies suggest that gastrodin possesses a range of neuroprotective effects. These include reducing inflammation, inhibiting the formation of harmful plaques associated with neurodegenerative diseases, and potentially improving cognitive function (Liu et al., 2018; Xue et al., 2023). Furthermore, research indicates an analgesic effect, suggesting gastrodin may be useful in managing chronic pain conditions, possibly by modulating specific cellular pathways involved in pain perception (Xiao et al., 2016). Despite its promise, gastrodin’s therapeutic potential remains under investigation. While preclinical studies have yielded positive results, further research is needed to fully understand its efficacy and safety in humans.

In this study, we used gastrodin, which has been proven to have multiple pharmacological effects, to conduct *in vitro* and *in vivo* experiments to explore its effects on valve calcification. The results showed that in VICs *in vitro*, gastrodin could significantly alleviate the upregulation of calcification indicators induced by osteogenic medium. In the *in vivo* high-fat-fed ApoE^−/−^ mouse valve calcification model, the addition of gastrodin could also significantly alleviate valve calcification. Through multi-omics analysis including RNAseq and metabolomics, it was speculated that the relief of VICs and heart valve calcification by gastrodin may be through regulating glycolysis pathway to regulate H3 histone lactylation, thereby inhibiting the osteogenic differentiation and calcification of VICs and heart valves, and exerting an anti-calcification effect.

## 2 Methods

### 2.1 VICs cell culture and treatment

VICs were cultured in high-glucose Dulbecco’s Modified Eagle Medium (DMEM) supplemented with 10% fetal bovine serum (FBS) and 1% penicillin-streptomycin (Pen/Strep). Osteogenic medium (OM, Stem Cell Technologies Cat No. 05465) was continuously treated for 12 days to induce osteogenic differentiation of VICs. Gastrodin (Selleck Chemicals, Cat. No. S2383) served as the experimental drug, while andrographolide (AGP, Selleck Chemicals, Cat. No. S2261) functioned as the positive control. VICs were treated with gastrodin at concentrations of 50 and 100 μM for 48 h *in vitro* experiments. Gastrodin is administered at a dose of 10 and 30 mg/kg *in vivo* using a high-fat diet-induced mouse model of CAVD.

### 2.2 Evaluation of VICs calcification induction and gastrodin intervention

VICs were seeded in 24-well plates at a density of 100,000 cells per well in 500 μL of complete culture medium containing 10% FBS and 1% penicillin-streptomycin (Pen/Strep). Each treatment group was set up with three replicate wells. After approximately 12 h of incubation, the culture medium was replaced with OM for the calcification induction group and OM supplemented with gastrodin or andrographolide for the intervention groups. The medium was changed every other day for 18 days. Subsequently, the cells were stained with Alizarin Red S to assess the degree of calcification and the effects of gastrodin and andrographolide intervention). The stained images were analyzed and quantitatively evaluated using ImageJ software.

### 2.3 RT-qPCR

Real-time quantitative PCR (RT-qPCR) was employed to evaluate the expression of osteogenic markers in VICs subjected to osteogenic induction and chemical intervention. Following an 18-day induction period, Cells in 24-well plates were washed three times with pre-cooled PBS, aspirated, and supplemented with 1 mL Trizol Reagent (Invitrogen, Thermo Fisher Scientific, Cat# 15596026CN). RNA was extracted using chloroform and precipitated with ethanol. RNA integrity and quantity were assessed, and 1 μg of total RNA was reverse transcribed into cDNA. RT-qPCR was then performed using SYBR Master Mix (TaKaRa Cat# RR820A) and specific primers for osteogenic markers. Data were collected on a Bio-Rad CFX96 instrument and analyzed using OriginPro software to determine gene expression levels. The osteogenic-related markers evaluated in the experiment and their corresponding RT-qPCR primers are listed in table 1.

**TABLE 1:** RT-qPCR Primers used in the project.

Marker	Forward Primer (5′-3′)	Reverse Primer (5′-3′)
RUNX2	CCC​AGT​ATG​AGA​GTA​GGT​GTC​C	GGG​TAA​GAC​TGG​TCA​TAG​GAC​C
COL1A2	CCT​GGT​GCT​AAA​GGA​GAA​AGA​GG	ATC​ACC​ACG​ACT​TCC​AGC​AGG​A
SPP1	CGA​GGT​GAT​AGT​GTG​GTT​TAT​GG	GCA​CCA​TTC​AAC​TCC​TCG​CTT​TC
BMP2	TGT​ATC​GCA​GGC​ACT​CAG​GTC​A	CCA​CTC​GTT​TCT​GGT​AGT​TCT​TC
PKM2	ATG​GCT​GAC​ACA​TTC​CTG​GAG​C	CCT​TCA​ACG​TCT​CCA​CTG​ATC​G
LDHA	GGA​TCT​CCA​ACA​TGG​CAG​CCT​T	AGA​CGG​CTT​TCT​CCC​TCT​TGC​T
LDHB	GGA​CAA​GTT​GGT​ATG​GCG​TGT​G	AAG​CTC​CCA​TGC​TGC​AGA​TCC​A

### 2.4 Western blotting

Western blotting was employed to evaluate the expression of osteogenic marker proteins in VICs under osteogenic induction and small molecule intervention. Cells were induced for 18 days and washed three times with PBS. After aspiration, 500 μL RIPA lysis buffer was added, and the cells were thoroughly scraped with a cell scraper. The lysates were transferred to centrifuge tubes and centrifuged at 4°C for 10 min. The supernatants were collected and mixed with one-sixth volume of protein sample buffer and denatured in a boiling water bath for subsequent use. SDS-PAGE was used to separate total proteins, which were then transferred to a PVDF membrane. The membrane was incubated with primary antibodies (all from Santa Cruz Biotech) working solution at 4°C overnight, followed by HRP secondary antibody (Santa Cruz Cat# sc-2357) incubation at room temperature for 2 h. The proteins were visualized using ECL reagents (From Thermo Fisher), and images were acquired. The relative quantification of protein bands was performed using Bio-Rad Quantity One software, and the data were analyzed using OriginPro.

### 2.5 In-cell western assay

The In-cell Western assay procedure was performed according to the manufacturer’s protocol (Xu et al., 2023). VICs were seeded into 96-well plates at an appropriate density and incubated until they reached approximately 70% confluency. Subsequently, cells were treated with different experimental formulations for 48 h. After treatment, the culture medium and treatment solutions were removed, and cells were fixed with paraformaldehyde and permeabilized with Triton X-100. The wells were then blocked with the manufacturer’s blocking buffer for 1 h at room temperature. Following blocking, primary antibodies were added and incubated overnight at 4°C. Subsequently, secondary antibodies were added and incubated for 1 h at room temperature in the dark. After several washes with PBST, the blots were imaged and analyzed using an Odyssey imaging system.

### 2.6 RNA-seq

In this study, RNA sequencing combined with bioinformatics analysis was employed to investigate the changes in gene expression profiles of VICs under different treatments. Cultured VICs were divided into three groups: a normal culture group, an osteogenic induction group, and an osteogenic induction followed by gastrodin treatment group. Total RNA was extracted using a commercial RNA extraction kit (Promega cat# LS1040) and sequenced by BGI (Shenzhen, China). After filtering out low-quality reads, the raw data were analyzed using R programming language for differential gene expression analysis and functional annotation, including Gene Ontology (GO) and Kyoto Encyclopedia of Genes and Genomes (KEGG) pathway enrichment analysis.

### 2.7 GC-MS metabolome analysis

The cell samples were washed with phosphate-buffered saline (PBS) and collected in methanol on ice. The collected cell samples were subjected to three freeze-thaw cycles using liquid nitrogen, followed by centrifugation at 12,000 rpm for 10 min at 4°C to obtain the supernatant. The supernatant was dried with nitrogen at 35°C, then treated with 80 μL of methoxypyridine solution (20 mg/mL) and 80 μL of N,O-bis(trimethylsilyl)trifluoroacetamide (BSTFA). The mixture was vortexed, centrifuged, and incubated in a water bath at 80°C for 1 h. After another round of centrifugation at 12,000 rpm for 10 min at 4°C, the supernatant was separated and stored for further analysis. One microliter of each prepared sample was injected into a gas chromatography system operating in electron ionization mode with an energy setting of −70 eV. Mass spectral data were acquired in full scan mode, covering mass-to-charge ratios (m/z) in the range of 50–650, consistent with previous literature.

### 2.8 Animal modelling for CAVD and assessment

All animal experiments involved in this study were approved by the Ethics Committee of Hubei University of Chinese Medicine (Approval No. 20220827009). 18 three-week-old ApoE^−/−^deficient SPF-grade mice were purchased from Huazhong University of Science and Technology (Animal License No. SCXK (E) 2021-0009 and No. SYXK (E) 2021-0057). The ApoE^−/−^ mice were divided equally into 3 groups of 6 mice each. To induce aortic valve calcification, mice were fed a high-fat diet (containing 42% fat and 0.25% cholesterol) for 16 consecutive weeks. Subsequently, from the 17th week, mice were fed a normal diet (control group), high-fat diet (CAVD group), or a high-fat diet supplemented with 10 mg/kg and 30 mg/kg gastrodin (GAS treatment group) for 8 consecutive weeks. Then, the mice were sacrificed, and their heart valves were collected for histological sectioning and Von Kossa staining. The staining results were semi-quantitatively analyzed using ImageJ software. Small animal cardiac ultrasound was used to evaluate flow velocity around the aortic valve.

### 2.9 Molecular docking

Gastrodin and corresponding protein molecules were obtained from the PubChem Compound and PDB databases, respectively. These structures were preprocessed using OpenBabel, AutoDock, and PyMOL to ensure compatibility with subsequent calculations. Binding energies were subsequently determined using AutoDock. The processed ligand and protein files were visualized using PyMOL for further analysis.

### 2.10 Statistical analysis

The data from three independent experiments were collected and represented as mean ± standard deviation (SD). For RT-qPCR and Western blotting, β-actin was normalized for all significance analyses, including one-way analysis of variance (ANOVA), multiple comparisons, and t-tests, using Origin software. A value of *p* < 0.05 was considered statistically significant.

## 3 Results

### 3.1 Gastrodin alleviates the calcification trend of VICs caused by osteogenic induction

**FIGURE 1:**
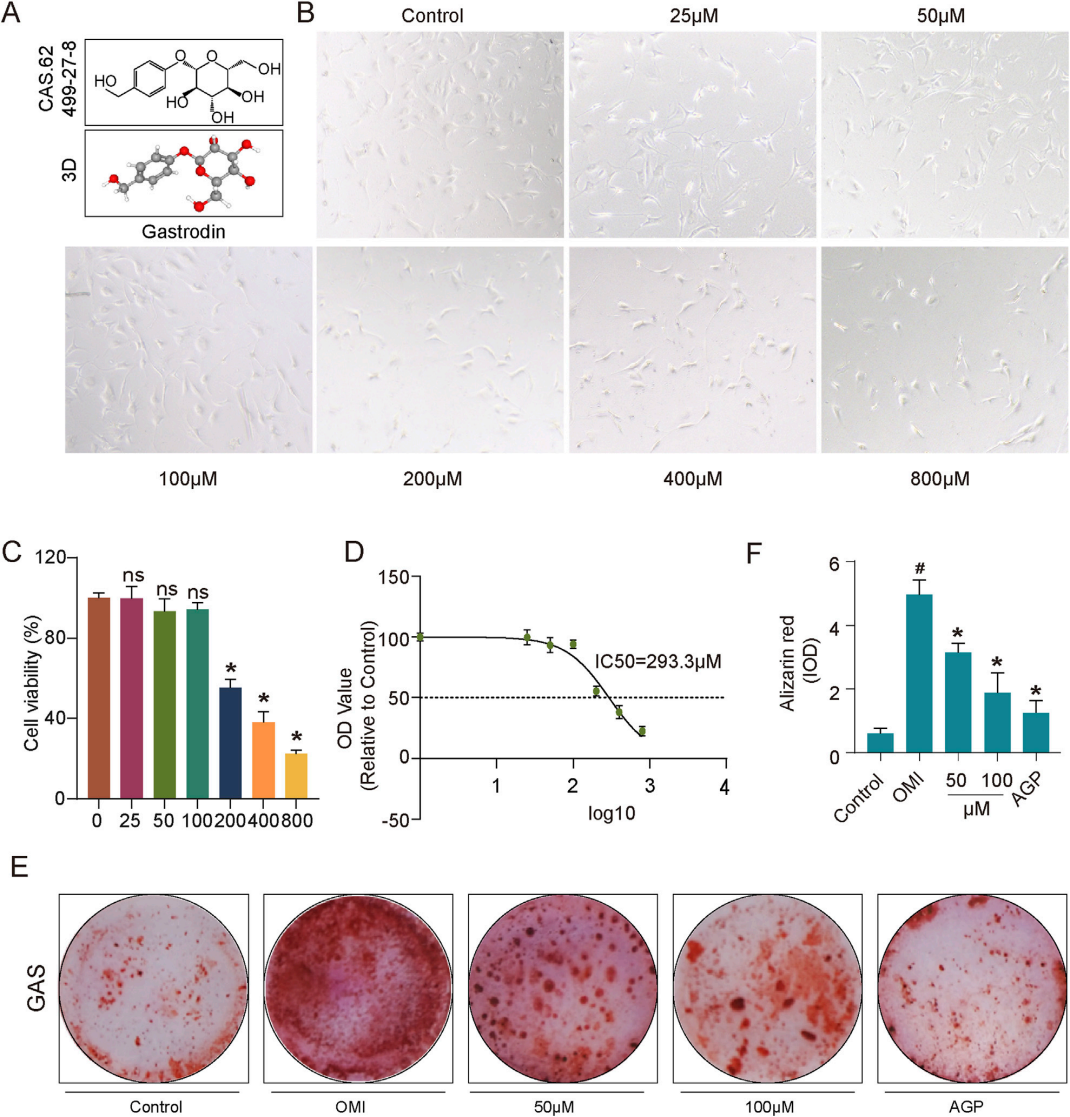
Impact of gastrodin (GAS) treatment on VICs viability and anti-calcification evaluation. **(A)** Molecular structure of GAS; **(B)** Microscopic photographs depicting growth of VICs under various treating concentration of GAS (n = 3); **(C)** Statistical analysis of GAS-treated VICs to assess the growth patterns and statistical significance of VICs treated with varying GAS concentrations (n = 3); **(D)** The IC50 of GAS on VICs; **(E,F)** Alizarin Red staining and semi-quantitative analysis: evaluating the calcification of VICs, OM-induced VICs (OMI-VICs), GAS-treated VICs, and AGP-treated VICs (n = 3). Statistical significance is indicated by # for OMI group vs control group and * for GAS or AGP group vs OMI group (*p* ≤ 0.05).

*Gastrodia elata* has a wide range of pharmacological activities, and its extract, gastrodin, has been used clinically for the treatment of cardiovascular diseases (Zhang, M. et al., 2023). To investigate the effects of gastrodin on aortic valve calcification, we conducted *in vitro* experiments using human valvular interstitial cells (VICs). Initially, VICs were treated with various concentrations of gastrodin to determine the optimal dosage (Figures 1A,B). Based on cell viability and CCK-8 assay results, the half-maximal inhibitory concentration (IC50) of gastrodin for VICs was determined to be 293.3 μM. However, cell viability was significantly reduced at 200 μM compared to the control group (Figures 1C,D). Therefore, 50 and 100 μM were selected as the working concentrations for subsequent experiments. To mimic calcification, VICs were cultured in osteogenic induction medium to promote osteogenic differentiation. Subsequently, these cells were treated with 50 or 100 μM gastrodin. AGP was used as a positive control to inhibit VICs osteogenic differentiation. Alizarin Red S staining was employed to assess mineralization. The results indicated that osteogenic medium significantly promoted VICs osteogenic differentiation, while gastrodin effectively suppressed this process (Figures 1E,F).

### 3.2 Gastrodin inhibits expression of calcification markers in VICs induced by osteogenic induction

**FIGURE 2:**
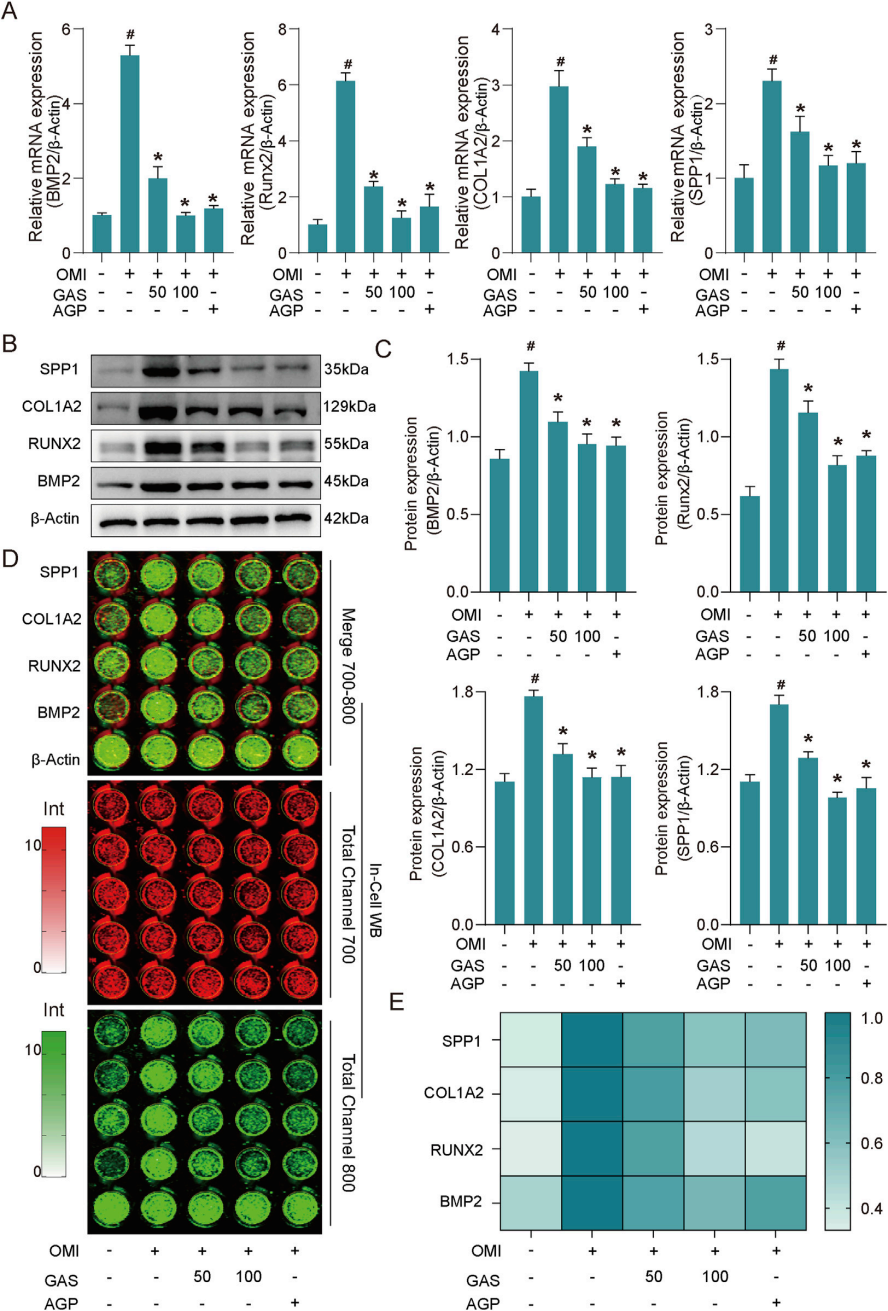
GAS inhibits the expression of osteogenesis-specific markers induced by OM. **(A)** RT-qPCR results show that GAS can significantly inhibit the expression of specific markers of osteogenesis in OM-induced VICs (OMI, OM-induced VICs group; GAS 50 μM treatment group; GAS 100 μM treatment group; AGP 10 μM treatment group) (n = 3). **(B)** Western blot results show that GAS can significantly inhibit the expression of specific markers of osteogenesis in OM-induced VICs (n = 3); **(C)** Semi-quantitative statistics of the band thickness of the Western blot results in B; **(D)** In-cell Western blot results show GAS can significantly inhibit the expression of osteogenic specific markers in OM-induced VICs (n = 3); **(E)** Semi-quantitative statistics of the intensity of In-cell blotting results in **(D)** Statistical significance is indicated by # for OMI group vs control group and * for GAS or AGP group vs OMI group (*p* ≤ 0.05).

To further validate these findings, the expression levels of osteogenic markers (BMP2, RUNX2, COL1A2, and SPP1) were evaluated at both the mRNA and protein levels and used AGP as a positive control (Wang, C. et al., 2024). Consistent with the Alizarin Red S staining results, RT-qPCR analysis revealed upregulated expression of these markers in osteogenically induced VICs, which was significantly inhibited by gastrodin and andrographolide treatment (Figure 2A). Western blotting further confirmed that the protein expression of these osteogenic markers was significantly increased in osteogenically induced VICs and was markedly suppressed by both concentrations of gastrodin and andrographolide, with a more pronounced inhibitory effect at 100 μM gastrodin (Figures 2B,C). The In-cell western assays further validated these conclusions (Figures 2D,E). The combined results of the RNA and protein expression analyses demonstrated that osteogenic induction medium successfully promoted VICs osteogenic differentiation *in vitro*, and gastrodin effectively inhibited this process.

### 3.3 Multi-omics analysis reveals that gastrodin alleviate the osteogenic differentiation of VICs by interfering glycolysis

**FIGURE 3:**
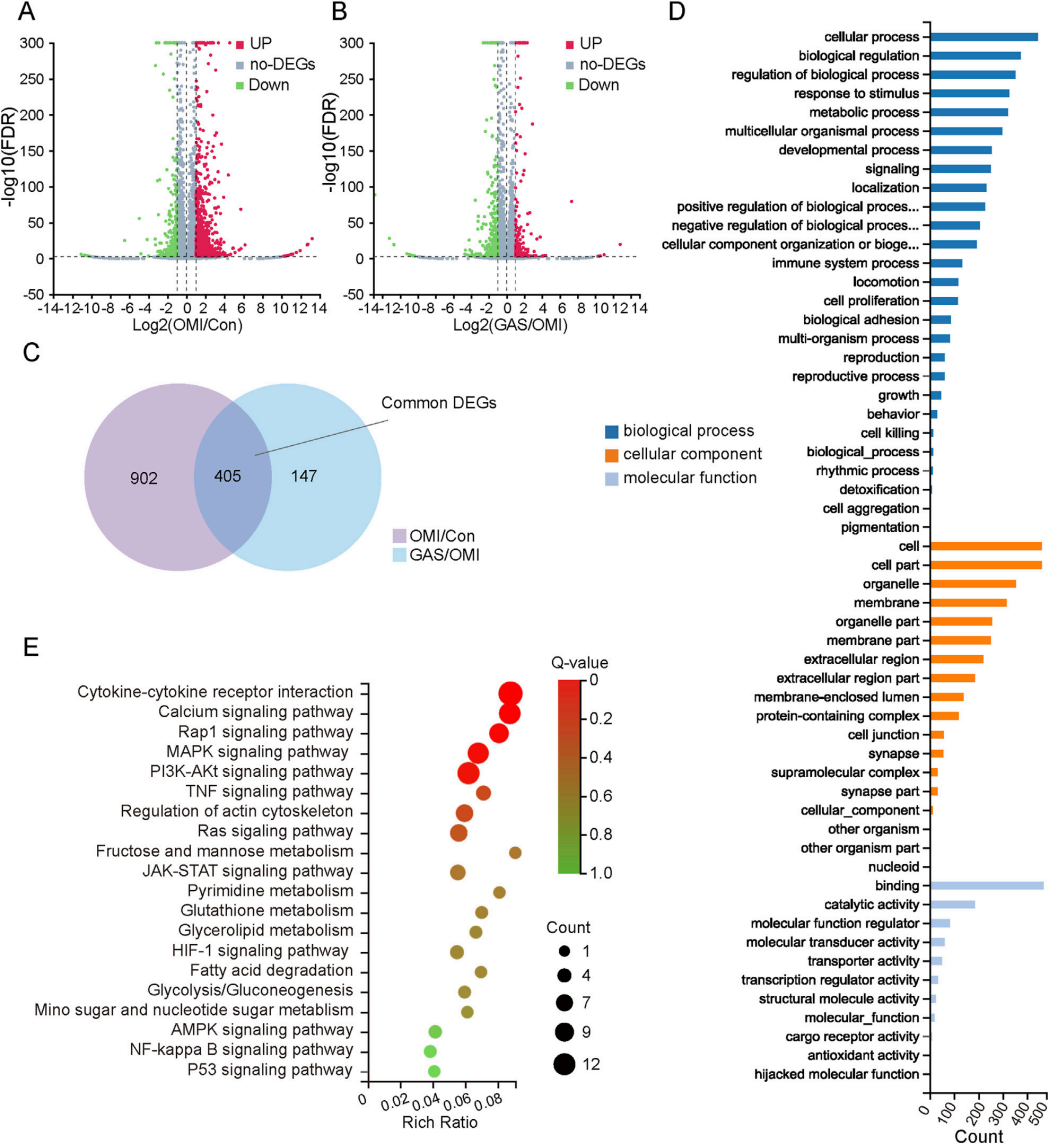
Gene expression profiles of mRNA sequencing with gastrodin (GAS) treatment under the condition of osteogenic medium induction (OMI). **(A,B)** Volcano map of differentially expressed genes (DEGs) in OMI versus control (OMI/Con), upregulation: 823 and downregulation: 484; in GAS versus OMI (GAS/OMI), upregulation: 247 and downregulation: 305, |log2 (fold change) (FC)| > 1 and false discovery rate (FDR) < 0.001 was considered as DEGs; **(C)** Venn interaction of DEGs of OMI/Con and GAS/OMI; **(D)** GO enrichment of the common DEGs including biological process, cellular component and molecular function; **(E)** KEGG pathway enrichment; a lower Q value indicates a higher degree of enrichment.

Previous findings demonstrated that the osteogenic induction medium successfully promoted osteogenic differentiation of VICs, and the addition of gastrodin significantly inhibited this process. To further elucidate the molecular pathways involved in gastrodin-mediated regulation of VICs osteogenesis, we conducted a comprehensive multi-omics analysis. Total RNA was extracted from three groups: VICs undergoing osteogenic induction, VICs treated with gastrodin and osteogenic induction medium, and untreated controls, with three biological replicates per group. RNA sequencing data were analyzed using R packages, revealing 823 upregulated and 484 downregulated genes in osteogenically induced VICs compared to controls (Figure 3A). Gastrodin treatment further induced 247 upregulated and 305 downregulated genes (Figure 3B). Venn diagram analysis identified 405 differentially expressed genes (DEGs) common to both comparisons (Figure 3C). Gene Ontology (GO) and Kyoto Encyclopedia of Genes and Genomes (KEGG) enrichment analyses revealed that these DEGs were primarily involved in cytokine signaling, metabolism, and calcium ion regulation (Figures 3D,E).

**FIGURE 4:**
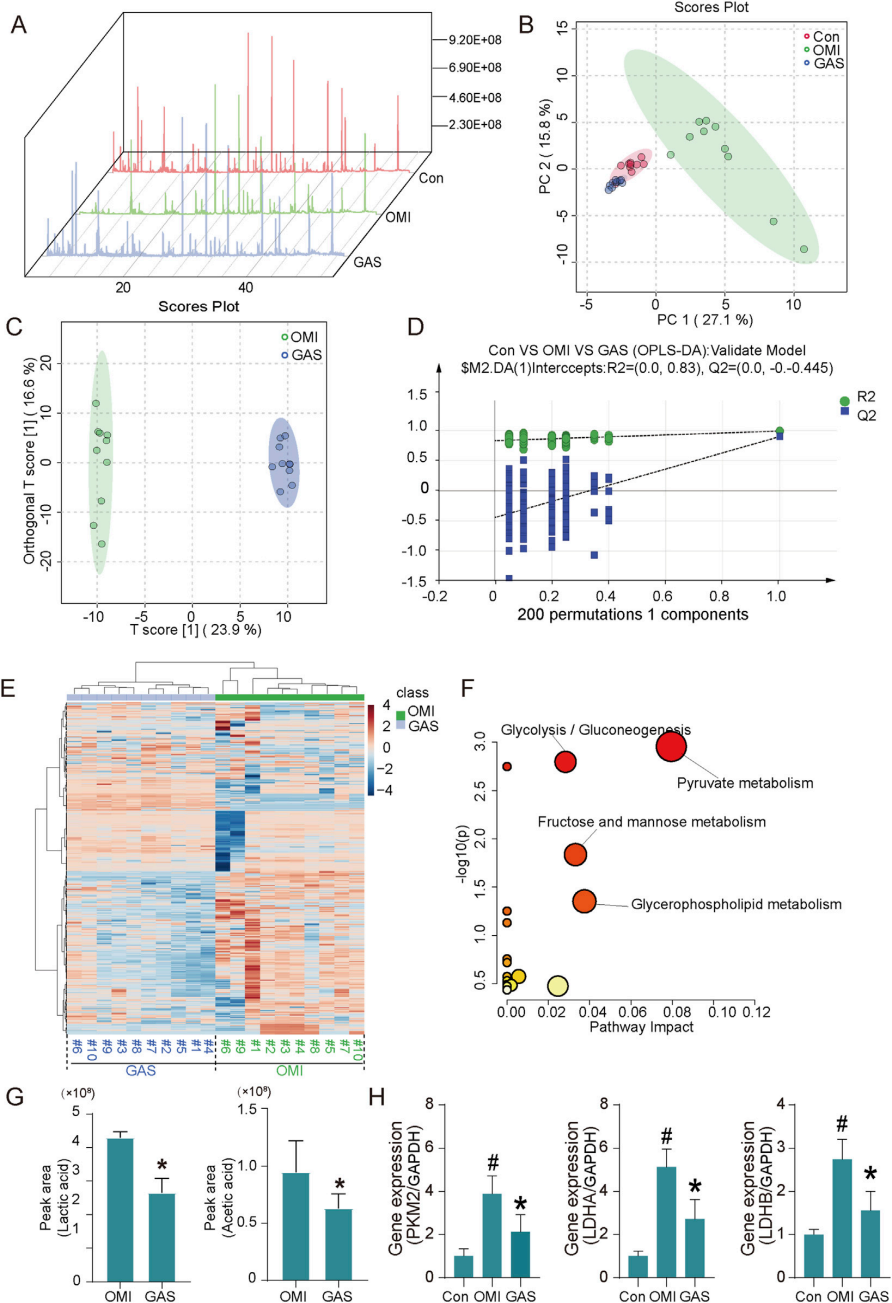
Metabolome analysis and expression evaluation of osteogenic medium-induced and gastrodin-treated VICs. **(A)** Total ion flow diagram of cell metabolites (n = 10); **(B)** Principal component analysis (PCA) diagram, **(C,D)** orthogonal partial least squares discrimination analysis diagram (OPLS-DA) (n = 10); **(E)** Cluster analysis of metabolome of OM-induced and GAS-treated VICs (n = 10); **(F)** Pathway enrichment based on differential metabolites (VIP>1), glycolysis/gluconeogenesis, pyruvate metabolism, fructose/mannose metabolism and glycerolipid metabolism are highlighted; **(G)** Top differential metabolite analysis including acetic acid and lactic acid, *p < 0.05 versus OMI (n = 10); **(H)** Glycolytic key gene expression detection of VICs with OM and OM plus GAS treatment (n = 3). #*p* < 0.05 versus control (con), *p < 0.05 versus OM.

To further investigate the metabolic pathways influenced by gastrodin treatment, we performed metabolomics analysis. The results showed significant differences in the metabolic profiles between the osteogenic induction group and the gastrodin-treated group (Figures 4A–D). Pathway enrichment analysis indicated that these differential metabolites were mainly enriched in glycolysis, pyruvate metabolism, and glycerolipid metabolism pathways (Figures 4D,E). Specifically, we observed a significant decrease in metabolites such as lactate, glycerol, and acetic acid in gastrodin-treated VICs (Figure 4G). The key glycolytic regulatory genes, LDHA, LDHB, and PKM2, were significantly downregulated in gastrodin-treated VICs compared to the control group (*P* < 0.05) (Figure 4H).

### 3.4 H3 histone lactylation plays a key role in gastrodin alleviating CAVD

**FIGURE 5:**
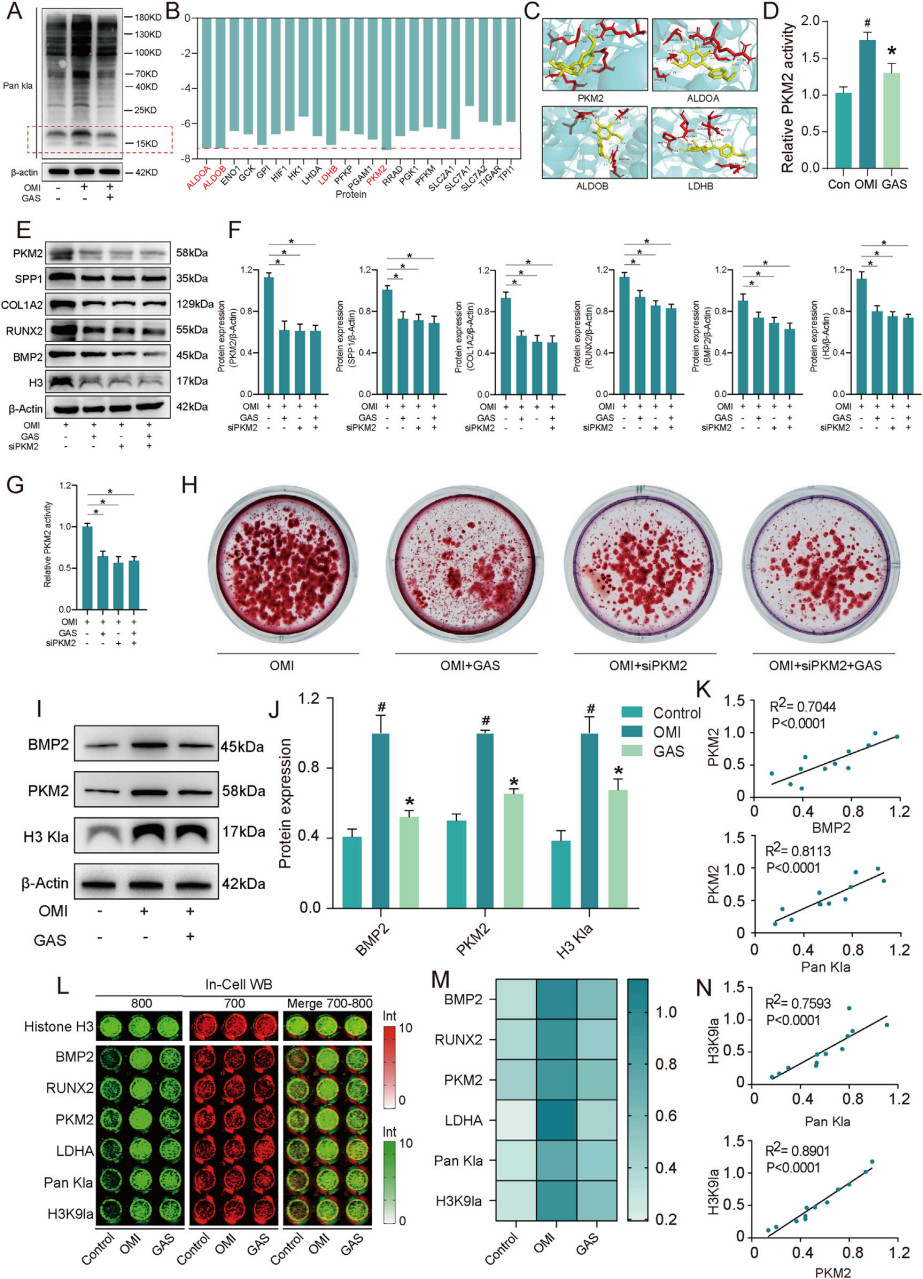
Gastrodin inhibits VICs calcification *via* H3 histone lactylation by interfering with PKM2. **(A)** Pan Kla expression levels of VICs with osteogenic medium (OM) and OM plus gastrodin (GAS) treatments (n = 3); **(B)** The binding energy of gastrodin and corelating proteins; **(C)** Molecular docking of gastrodin and the proteins with 4 least binding energy PKM2, ALDOA, ALDOB, LDHB; **(D)** Enzyme activity of PKM2 in VICs treated with gastrodin and untreated with gastrodin protein under OM induction (n = 3); **(E,F)** Expression levels of PKM2, SPP1, COL1A2, RUNX2, BMP2 and H3 Kla of VICs under OM inducing with gastrodin and siPKM2 treating (n = 3); **(G)** Enzyme activity of PKM2 in VICs under OM inducing with gastrodin and siPKM2 treating (n = 3); **(H)** Alizarin Red staining in VICs under OM inducing with gastrodin and siPKM2 treating (n = 3); **(I,J)** Expression levels of BMP2, PKM2 and H3 Kla of VICs under OM inducing with and without gastrodin treating (n = 3); **(K)** Correlation analysis of the expression data above; (**L,M)** In-cell Western blotting of BMP2, RUNX2, PKM2, LDHA, H3K9la of VICs under OM inducing with and without gastrodin treating, Histone H3 (n = 3); **(N)** Correlation analysis of the expression data above. #*p* ≤ 0.05 in OMI vs Control, *p ≤ 0.05 in GAS-treated vs OMI.

Having established that gastrodin’s inhibition of calcification is linked to lactate regulation, we identified lactate-induced histone lactylation as a pivotal mechanism underlying glycolysis-driven valve calcification. Initially, we observed a significant upregulation of lactylation levels in VICs following osteogenic induction, an effect that was attenuated by gastrodin. Notably, the proteins exhibiting the most pronounced alterations were approximately 15 kDa in size, consistent with the characteristic mass of H3 histones (Figure 5A). This suggested that gastrodin’s anti-osteogenic effects were likely mediated by altering H3 histone lactylation. Subsequently, we employed AutoDock software to compute the binding energies between gastrodin and the 23 proteins most strongly correlated with lactylation (Figure 5B). Molecular docking models were generated for the four proteins with the lowest binding energies (Figure 5C). Our results indicated that pyruvate kinase M2 (PKM2), a key glycolytic enzyme, is the most probable target of gastrodin, influencing the osteogenic differentiation of VICs. Gastrodin treatment significantly inhibited the enzymatic activity of PKM2 (Figure 5D). To verify the inhibitory effect of GAS on PKM2, we performed PKM2 knockdown experiments *in vitro*. The results showed that both GAS treatment and PKM2 knockdown significantly inhibited PKM2 expression. Both GAS treatment and PKM2 knockdown significantly inhibited the protein expression of these calcification-related markers, and also inhibited histone lacylation levels (Figures 5E,F). In addition, we observed no significant difference between the GAS-treated and siPKM2 groups, whereas the calcification-related markers in the GAS + siPKM2 group were lower than those in the GAS-treated and siPKM2 groups, but not significantly. The results of PKM2 activity (Figure 5G) and alizarin red staining (Figure 5H) experiments were consistent. These results suggest that GAS exerts an anticalcification effect by inhibiting PKM2. To corroborate these findings, we examined osteogenic markers, PKM2, and H3 histone lactylation levels in VICs subjected to osteogenic induction and gastrodin treatment. As expected, the expression levels of all three increased following osteogenic induction and were significantly suppressed by gastrodin (Figures 5I–K). Furthermore, using In-cell western assay, we analyzed a panel of osteogenic markers and H3 histone modification-related markers and performed correlation analyses. Intriguingly, PKM2, a molecule closely associated with gastrodin, exhibited the strongest correlation with the H3 histone lactylation marker lysine 9 (H3K9la) (Figures 5L–N). These results suggest that gastrodin may inhibit VICs osteogenic differentiation by interfering with H3 histone lactylation.

### 3.5 Gastrodin attenuates valve calcification in a mouse model of calcific aortic valve disease

**FIGURE 6:**
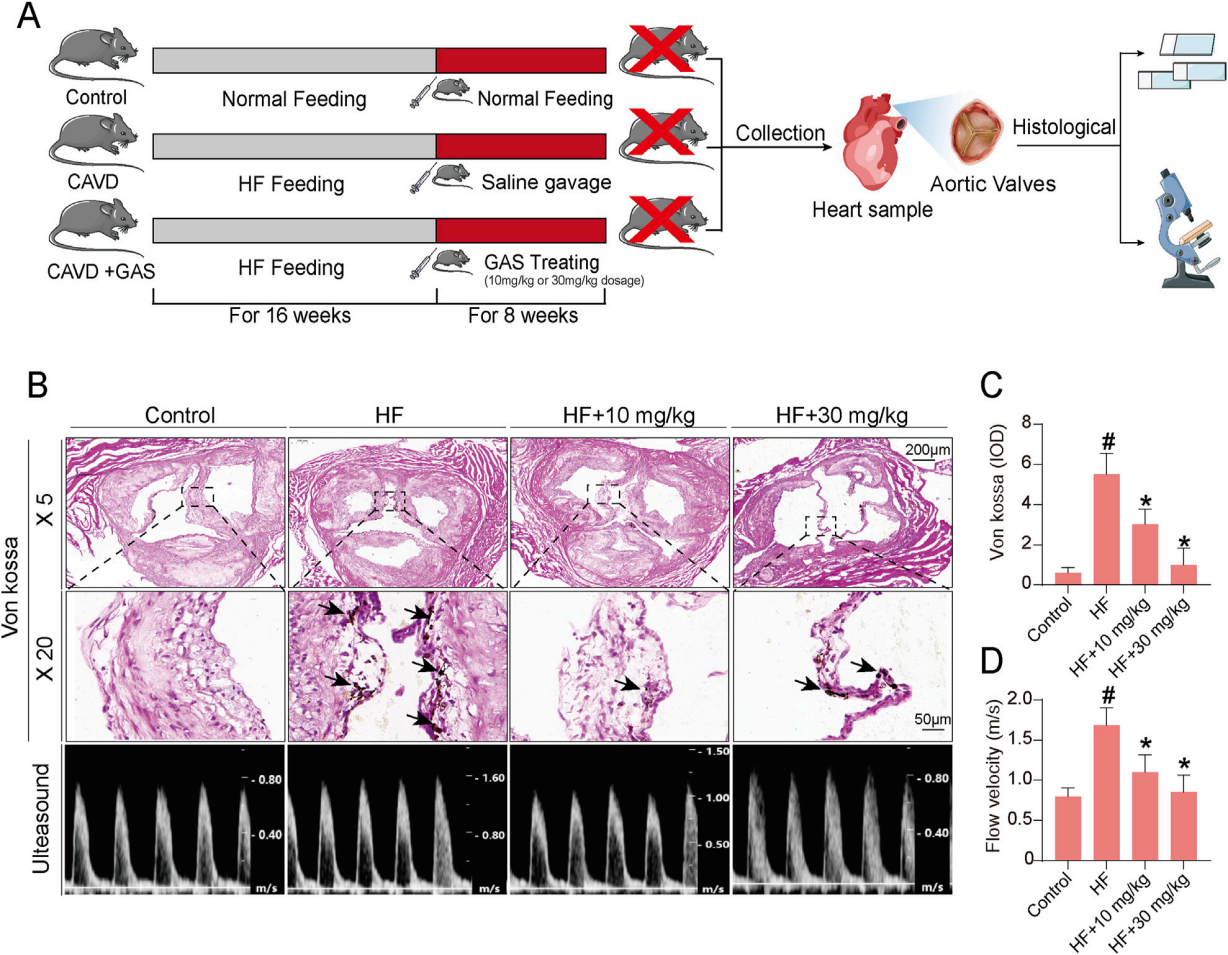
Gastrodin can alleviate calcification level of CAVD in mice. **(A)** The schematic diagram illustrates the validation of gastrodin accumulation in heart tissue involving in alleviating aortic valve calcification in the ApoE^−/−^ mice; **(B)** Calcification evaluating results of CAVD mouse model plus gastrodin addition by Von Kossa staining and cardiac ultrasound; **(C)** Statistics of Von Kossa staining; **(D)** Statistics of cardiac ultrasound. N = 6, #*p* < 0.05 versus control, *p < 0.05 versus HF.

Previous *in vitro* studies using VICs have demonstrated that osteogenic induction media can successfully induce osteogenic differentiation of VICs, and gastrodin can alleviate this osteogenic differentiation. However, whether gastrodin can alleviate aortic valve calcification *in vivo* remains unknown. In this study, we established a CAVD model by feeding ApoE^−/−^ mice a high-fat diet for 16 weeks. Subsequently, the mice were orally administered gastrodin for 8 weeks, and their aortic valves were harvested for histological analysis and Von Kossa staining to assess valve calcification (Figure 6A). The results showed that the high-fat diet induced CAVD in ApoE^−/−^ mice. Gastrodin administration significantly inhibited valve calcification, and a dose of 30 mg/kg was more effective in rescuing valve calcification in the CAVD mice model than 10 mg/kg (Figures 6B,C). The results of cardiac ultrasound are consistent with the results of Von Kossa staining (Figure 6D).

## 4 Discussion

CAVD is the third most common cardiovascular disease after coronary heart disease and hypertension, and its prevalence increases dramatically with age, with a prevalence of more than 25% in people over 65 years of age (Kraler et al., 2022).The pathogenesis of CAVD is complex and it is a chronic disease involving extracellular matrix deposition, fibrosis, inflammation and endothelial dysfunction (Tanase et al., 2022). There are currently no drugs on the market worldwide that can treat or delay CAVD, and surgical aortic valve replacement (SAVR) or transcatheter aortic valve replacement (TAVR) are the only options for patients with severe symptomatic CAVD (Moncla et al., 2023). It is therefore important to explore pharmacological interventions in the early stages of aortic calcification to prevent or slow the progression of CAVD.

In this study, the therapeutic potential of gastrodin, a natural compound isolated from the dried root mass of the traditional Chinese medicine *gastrodia elata blume*, in reducing aortic calcification was observed. We found that 50 and 100 μM of gastrodin significantly inhibited the expression levels of calcification markers SPP1, COL1A2, RUNX2, and BMP2 in VICs cells by RT-qPCR, Western blotting, and In-cell. To investigate the molecular mechanism by which gastrodin exerts its role in alleviating aortic valve calcification, the present study combined RNA-Seq and metabolomics analyses to confirm that gastrodin exerts its anti-calcification effects by targeting glycolysis. Oxidative phosphorylation (OXPHOS) is the main mode of energy metabolism within the cell, but under pathological conditions cells undergo metabolic reprogramming from oxidative phosphorylation to aerobic glycolysis (Feng et al., 2022). Studies have shown that glycolysis plays an important role in human energy metabolism, but its abnormalities can lead to the onset and progression of a variety of chronic diseases, such as chronic liver disease (Qu et al., 2023), cancer (Chelakkot et al., 2023) and cardiovascular disease (Chen et al., 2023). Our previous studies have also shown that glycolysis is involved in the calcification process of aortic valves (Huang et al., 2024). Therefore, targeting glycolysis may be a potential therapeutic avenue to treat or alleviate aortic valve calcification.

As a key enzyme in the glycolytic pathway, pyruvate kinase M2 (PKM2) plays an important role in the regulation of cellular functions (Zhang, Z. et al., 2019). At the metabolic level, it catalyzes the conversion of phosphoenolyruvate to pyruvate, which is an important promoter of aerobic glycolysis and promotes energy supply and biosynthesis in abnormal cells. At the level of transcriptional regulation, PKM2 enters the nucleus as a transcriptional co-activator and is involved in regulating the expression of genes related to cell growth and proliferation. PKM2 has been implicated in the development of a variety of cardiovascular diseases, including heart failure, myocardial infarction and pulmonary hypertension (Rihan and Sharma, 2023). A research team at the University of Iowa discovered the important role of PKM2 in regulating platelet function and thrombosis and identified a small molecule activator (ML265) that can inhibit platelet activation and thrombosis by stabilizing PKM2 tetramers while preventing the formation of PKM2 dimers and modulating the downstream PI3K/Akt/GSK3 signaling pathway (Nayak et al., 2021). Using molecular docking and other experiments, we found that gastrodin targets and inhibits the enzymatic activity and protein expression of PKM2, which in turn affects the glycolysis level of VICs.

Lactate, the end product of glycolysis, has long been neglected as a metabolic waste product of glucose metabolism under hypoxic conditions (Rabinowitz and Enerbäck, 2020). There is increasing evidence that lactate is a universal metabolic fuel for tissues such as skeletal muscle, heart, brain and tumor cells, contributing to cell fate decision processes (Lin et al., 2022). It is also thought to act as a metabolic buffer, linking glycolysis and oxidative phosphorylation. In addition, lactate can act as a signaling molecule for a variety of regulatory functions such as immune cell regulation, lipolysis, wound healing and maintenance of cellular homeostasis. Previous studies have shown that the accumulation of lactate from glycolysis promotes the progression of aortic valve calcification by increasing osteogenic differentiation of the VICs (Wang C. et al., 2024). The present study showed that gastrodin could inhibit the glycolysis level of VICs through inhibiting the enzyme activity and protein expression of PKM2 and thus reduce their lactate production level.

Lactate is an essential metabolite in transcriptional regulation, and Professor Yingming Zhao’s team revealed the histone lysine lactylation using lactate as a substrate (Zhang, D. et al., 2019). Histone lactylation, an indicator of glycolysis and lactate levels, is intrinsically linked to cellular metabolism and represents a new epigenetic code that influences cell fate and plays important functions in inflammation (Irizarry-Caro et al., 2020), neurological diseases (Wang X. et al., 2024), pulmonary fibrosis (Wang P. et al., 2024) and tumors (Feng et al., 2024). In addition, lactylation has been shown to play an important role in the pathogenesis of cardiovascular diseases such as myocardial infarction (Wang et al., 2022), heart failure (Zhang, N. et al., 2023), atherosclerosis (Wang et al., 2023) and ischaemic stroke (Zhou et al., 2024) by affecting protein function and gene expression. Notably, our published studies have shown that H3 histone lactylation plays an important role in driving CAVD calcification, and that reduction of histone lactylation by 2-DG or LDHA knockdown inhibits calcification in VICs, demonstrating a direct link between H3 histone lactylation and calcification (Huang et al., 2024). Our further studies revealed that gastrodin inhibits the level of histone lactylation of VICs by diminishing their glycolysis level. Notably, the level of H3 lactylation in VICs was positively correlated with the expression of the calcification marker BMP2.

However, this study has several limitations. First, the differentially expressed genes in VICs in the presence or absence of gastrodin were enriched in several inflammation-related pathways, suggesting that gastrodin may also potentially exert an anti-calcification effect through anti-inflammatory properties, which requires further analysis of the transcriptomic data. Second, this study could not precisely identify PKM2 in VICs as a molecular target of gastrodin *in vivo*, which may require the construction of transgenic mice with specific knockout of PKM2.

In conclusion, gastrodin attenuates aortic valve calcification by inhibiting histone lactylation through PKM2 regulation of the glycolytic pathway. These findings highlight the potential therapeutic application of gastrodin in the treatment of CAVD.

## Data Availability

The original contributions presented in the study are publicly available. This data can be found here: NCBI BioProject (PRJNA1173779).
